# Primary Aorto-Enteric Fistula With a Subsequent Secondary Aorto-Enteric Fistula

**DOI:** 10.1016/j.ejvsvf.2024.05.005

**Published:** 2024-05-14

**Authors:** Iris Kärjä, Venla Soini, Olli Hautero, Maarit Venermo

**Affiliations:** a Department of Surgery, Vaasa Central Hospital, Wellbeing Services County of Ostrobothnia, Vaasa, Finland; b Department of Pediatric Surgery, University of Turku and Turku University Hospital, Turku, Finland; c Department of Vascular Surgery, Abdominal Centre, University of Helsinki and Helsinki University Hospital, Helsinki, Finland; d University of Helsinki, Helsinki, Finland

**Keywords:** Abdominal aorta, Aorto-enteric, Digestive system fistula, Gastrointestinal bleeding, Primary aorto-enteric fistula, Secondary aorto-enteric fistula

## Abstract

**Objective:**

Primary aorto-enteral fistula (PAEF) is a connection between the gastrointestinal tract and the aorta that occurs without previous aortic surgery. The aetiological factors include, but are not limited to, aneurysm, infection, and tumours. It is a life threatening condition if untreated and requires emergency vascular surgical repair. A secondary aorto-enteric fistula (AEF) can occur to a previously reconstructed aorta. This case report presents a unique case of a male patient who developed a primary AEF and subsequent secondary AEF with successful surgical outcomes, suggested to be due to tuberculous aortitis.

**Report:**

The patient was diagnosed and treated for tuberculosis and developed a saccular aneurysm within six months. The PAEF was surgically corrected with a tube graft using a bovine pericardial patch, the defect in duodenum was sutured, and a retrocolic omental flap was created between the duodenum and aorta. He developed a small stable pseudoaneurysm during follow up, and then a secondary AEF two and a half years later, in which a connection between the pseudoaneurysm and duodenum was corrected using a new bovine aorto–aortic interposition graft using a bovine pericardium patch. The defect in the duodenum was also sutured in two layers and a new omental flap was created.

**Discussion:**

The mortality rate of AEF is high and it is very unlikely that a patient will survive two AEFs without major complications. It is believed that there are extremely few double AEF cases described in the literature. The aetiological factor in the development of PAEF in this case was most likely the patient's aortic aneurysm, which was most likely of mycotic origin due to tuberculosis. The patient developed a pseudoaneurysm during follow up and it is uncertain whether the pulsatile pressure of the pseudoaneurysm led to the recurrence of the AEF.

## INTRODUCTION

Primary aorto-enteric fistula (PAEF) is a communication between the native aorta and gastrointestinal (GI) tract, while the communication is formed between the GI tract and previously reconstructed aorta in the secondary form.^1^ The overall incidence of PAEF is very low, at approximately 0.05%,^2^ and recurrence of AEF after aortic repair is rare.^3^ The aetiological factors for PAEF include aortic aneurysm, tumours, radiotherapy, and infection. In > 50% of cases, the condition affects the distal third of the duodenum.^4^ Primary AEF based on tuberculous aortitis has been described previously in the literature.^5^ Secondary AEF is most often caused by mechanical failure or infection of the stent graft, but bowel and graft adhesions also play an undeniable role in fistula development.^6^^,^^7^ The classical triad of symptoms for AEF include GI bleeding, abdominal pain, and a pulsatile abdominal mass.^8^ The treatment of AEF depends on the aetiology and clinical situation of the patient and includes patient stabilisation, rapid aortic control, and definitive fistula repair.^9^

## CASE REPORT

**Figure 1:**
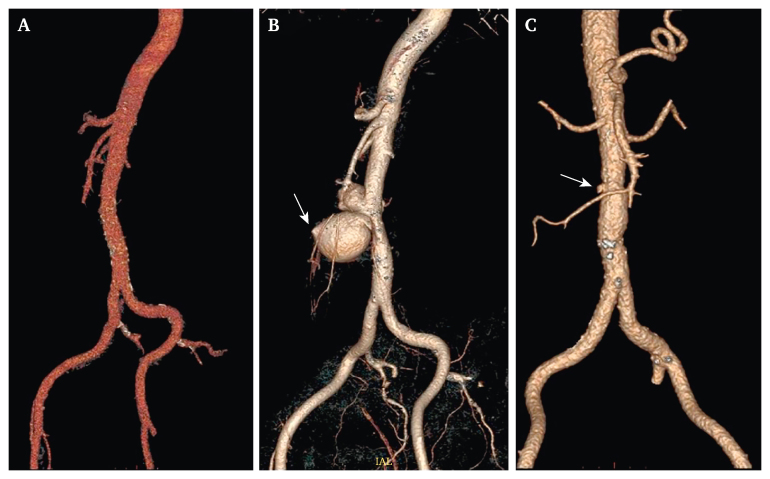
Three dimensional reconstructions of the evolution of the double aorto-enteric fistula (AEF). (A) A reconstruction of the aorta six months prior to the primary aorto-enteric fistula. (B) Imaging taken at the clinical presentation of the primary AEF; the scan shows a clear saccular aneurysm with extravasation to the duodenum (arrow). (C) Pseudoaneurysm (arrow) on the latest computed tomography scan available two weeks pre-operatively for the secondary AEF (focused on the latter secondary AEF).

The patient was an 80 year old male, non-smoker, with arterial hypertension, prostatic hyperplasia, hypothyroidism, sleep apnoea, and meningioma. The patient had no history of GI bleeding or aortic disease. He was diagnosed with hairy cell leukaemia in July 2019. In March 2020, full body computed tomography (CT) imaging was performed to determine the leukaemia stage, without findings of aortic pathology (Fig. 1A).

During the leukaemia treatment, sarcoidosis was suspected due to pathological lymph nodes and spleen changes; however, the symptoms persisted despite adequate treatment, and broncho-alveolar lavage was performed. Surprisingly, the patient was diagnosed with tuberculosis (TB). Nine months of antibiotic treatment (ethambutol, pyrazinamide, rifampicin, and isoniazid for the first two months followed by rifampicin and isoniazid treatment for the following 7 months) was initiated in June 2020.

**Figure 2:**
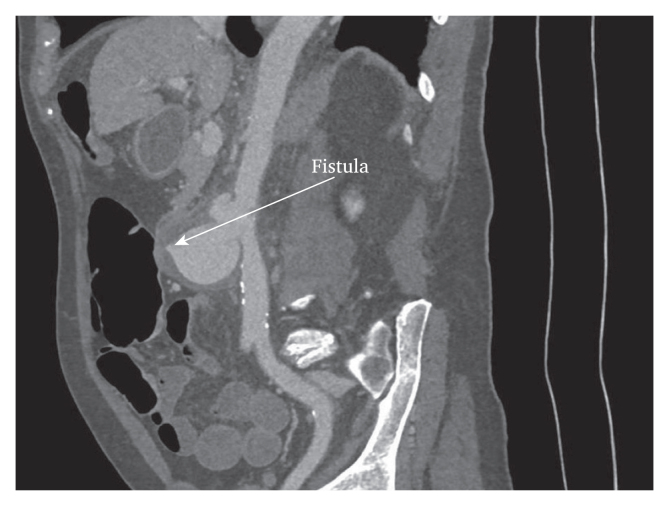
Multiple planar reconstruction of a computed tomography scan showing a large aortic aneurysm and primary aorto-enteric fistula (arrow).

The patient presented to the emergency department in September 2020 with anaemia, melaena, and stomach pain. Gastroscopy and colonoscopy were performed: diverticular disease and a few polyps were found. The patient was discharged as he was stable. Later that same day he suddenly felt nauseous, collapsed with seizure, and was re-admitted to the emergency department haemodynamically unstable. Coagulated blood in the stomach and a duodenal ulcer were found during emergency gastroscopy, but there were no signs of active bleeding. As melaena and hypotension persisted, CT angiography was performed and a 6.1 cm diameter infrarenal saccular aortic aneurysm with suspected AEF was diagnosed (Figure 1, Figure 2, arrows) and the patient underwent an emergency operation.

During successful surgery, a tube graft was created from a bovine pericardial patch. The aneurysm sac was incised, revealing a fistula to the duodenum at ligament of Treitz level; a mycotic aneurysm was suspected. The duodenum was sutured and a retrocolic omental flap was transferred and fixed between the aorta and duodenum. A full thickness aortic wall tissue sample was taken and interpreted as non-specific vasculitis. Bacterial cultures were negative. The patient received additional intravenous antibiotic therapy until the sixth post-operative day, which was continued orally for another 10 days. He was initially treated with cefuroxime and metronidazole, after which he developed an acute generalised skin reaction. The antibiotics were changed to vancomycin and meropenem, followed by oral moxifloxacin according to infectious diseases specialist advice. He then continued on TB treatment until February 2021. The suspected antibiotic reaction was later diagnosed as an allergy to the CT contrast agent. Consequently, magnetic resonance angiography (MRA) was favoured for the follow up scans. In May 2021, a small 9 x 6 mm pseudoaneurysm at the proximal anastomotic seam was discovered on a follow up MRA. The size of the pseudoaneurysm remained constant in repeated scans and no further action was taken.

**Figure 3:**
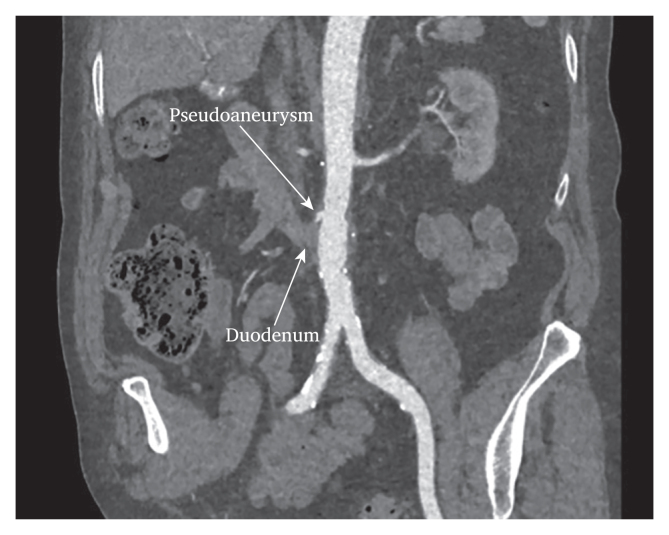
Multiple planar reconstruction of the computed tomography angiography scan two weeks pre-operatively for the secondary aorto-enteric fistula, showing a clear adhesion (arrow) between the duodenum and aorta.

In February 2023, the patient had a ward stay due to anaemia and fainting, during which colonoscopy, gastroscopy, and contrast enhanced CT scan were performed on vital indications, resulting in a severe allergic reaction. The examinations showed no evidence of active leakage or AEF. Retrospectively, the presence of recurrent AEF might have been suspected due to contact between the duodenum and pseudoaneurysm (Fig. 1C, Fig. 3, arrows).

In March 2023, the patient was admitted to the surgical ward due to fainting and melaena, with a haemoglobin of 70 g/L. Gastroscopy again revealed bloody gastric content without active leakage, which raised the clinical suspicion of a recurrent AEF. A non-contrast CT scan showed the distal part of the duodenum to be adherent to the aorta. An emergency exploratory laparotomy was performed, and recurrent AEF was found originating from the pseudoaneurysm. During successful surgery, the duodenum was detached from the aorta and the defect was sutured over in layers. The previous graft was changed to a new aorto–aortic interposition graft using bovine pericardium. In addition, a new omental flap was placed between the aortic reconstruction and duodenum to protect the graft and prevent further fistulation.

Two months post-operatively, the patient was doing well. The MRA imaging showed aortic dissection proximal to the graft that was probably caused by intra-operative clamping. A surveillance approach was taken in this regard. The patient provided written consent for the publication of this case report.

## DISCUSSION

This case report presents an extremely rare case of a patient with both primary and secondary AEF. The AEF mortality rate is high and it is very unlikely that a patient will survive two AEFs without major complications. It is believed that only a few AEF cases have been described in the literature. Tabbara et al. described a similar double AEF case with patient survival with aetiology of chronic Q fever^10^ and Jiber et al. a double AEF situation at the onset of Behçet's disease.^11^

The primary aetiological factor in the development of PAEF in this case was probably the abdominal aortic aneurysm, but examinations also raised suspicions of aortitis due to TB, which is a possible cause of mycotic aneurysm and AEF.^12^ Earlier case reports have also described AEF with TB origin.^5^^,^^13^ The Consultant Rheumatologist suspected that the former LITAK treatment for hairy cell leukaemia activated a latent form of TB, which might have enhanced the development of the abdominal aortic aneurysm. The suggestion of a TB origin underlying the patient's PAEF is also supported by the fact that the aneurysm was saccular rather than fusiform, with a detectable inflammatory rim on imaging. Furthermore, the aneurysm developed rapidly, as a full body CT scan taken six months earlier showed no signs of aortic pathology, even on retrospective examination. In addition, it should be noted that the TB treatment, started a few months earlier, may have caused the TB to subside to the extent that it no longer showed up in bacterial culture samples.

A graft infection is known to be a crucial cause of a secondary AEF in the literature.^8^ In the case of a mycotic aneurysm, it should be noted that surgery is only part of the treatment and the need for antimicrobial therapy should be carefully assessed. Infection can lead to the formation of a pseudoaneurysm, which this patient had on follow up scans. As he underwent the primary operation with an allograft rather than a prosthesis, it is uncertain whether the pulsatile pressure of the pseudoaneurysm led to recurrence of the AEF. It is important to acknowledge that biological graft material has a risk of infection and failure, although biological graft material, including pericardial patch, has a very low re-infection rate after mycotic aneurysm repair^.^^14^ Furthermore, the tissue samples taken during secondary AEF surgery showed no signs of infection.

### Conclusion

In conclusion, this patient was diagnosed with primary and later secondary AEF, both of which were treated successfully. The literature concerning dual AEF is extremely limited, which makes this case particularly interesting.

## Conflict of interest

None.

## FUNDING

VS has received personal research grants from Vappu Uuspään säätiö, the Finnish Paediatric Research foundation, and Turku University research funding. OH has received a personal research grant from Jussi Lallin ja Eeva Mariapori-Lallin säätiö and the Finnish Research foundation. MV has received a lecture fee from Abbot. IK has received a personal research grant from Vaasan Lääketieteellinen säätiö. This research did not receive any specific grant from funding agencies in the public, commercial, or not for profit sectors.
